# Supplementary material for: WordCommentsAnalyzer: A windows software tool for qualitative research
Source: F1000Res. 2018 Sep 4;7:536. Originally published 2018 May 3. [Version 2] doi: 10.12688/f1000research.14819.2 (PMC6137411; doi:10.12688/f1000research.14819.2)
Supplement: Supplementary file 3 [file f1000research-7-17614-s0002.tgz › b2a79496-4ada-47c4-9682-a99ce867836d.zip!/Eight Iranian Health Journals/Journal of Knowledge and Health_37.docx]

© Journal of Knowledge and Health. This work is licensed under CC BY 4.0 (https://creativecommons.org/licenses/by/4.0/).

Volume & Issue: دوره 4، شماره 1: 1388

Title: اندازه¬گیری شاخص¬های تنفسی در جمعیت¬های نمونه مناطق مختلف ایران و تعیین معادله های مرجع به منظور برآورد این شاخص ها در افراد ایرانی

http://knh.shmu.ac.ir/index.php/site/article/view/176

مقدمه: مقادير طبیعی پارامترهاي حياتي قلبي، ريوي و یا خونی در جمعیت­های مختلف یکسان نبوده بلکه تحت تأثير متغيرهايي مانند سن، جنس، قد، ژنتيك و موقعیت جغرافیایی قرار مي­گيرند. هدف از اين تحقيق تعيين اندازه­هاي فيزيولوژيك پارامترهاي تنفسی در جمعيت طبیعی ايران و به­دست آوردن معادلات مرجع براي اين گروه می­باشد. مواد و روش­ها: افراد نمونه از شهرهاي مختلف انتخاب و پس از اطمينان از سلامت آن­ها براي تست به آزمايشگاه اعزام شده و در آزمايشگاه پارامترهاي حياتي ریوي شامل حجم جاری (TV)، ظرفیت حیاتی سریع (FVC) و آهسته (SVC)، تعداد تنفس (RR) و حجم جریان بازدمی (FEV) با شيوه­هاي استاندارد و رعايت اخلاق پزشكي در جنس­هاي زن و مرد به­طور جداگانه اندازه­گیری شد. شرايط آزمايشگاه براي همه افراد يكسان و در حالت نشسته و پس از استراحت بوده است. نتایج: نتايج حاصل از اين اندازه­گيري­ها در گروه­هاي سني مختلف و به تفكيك زن و مرد تعيين شد. پس از تعيين ميانگين براي گروه­هاي سني و با استفاده از فرمول­های رگرسيون، معادلات مرجع براي هر كدام از پارامترهاي حياتي مورد نظر به شرح زير به­دست آمد. (قد)003/0+(جنس)031/0+035/0- =TV، (قد)020/0+(سن)004/0-651/0- =FEV1، (وزن)035/0-(قد)108/0-(سن)033/0-791/44 =RR، (قد)019/0+424/0- =SVC، (وزن)003/0+(قد)010/0+(جنس)083/0+842/0 =FVC. نتیجه­گیری: با توجه به معادلات مرجع به­دست آمده براي جمعيت سالم ايران، مي­توان با در دست داشتن خصوصيات فرد مانند، قد، وزن و سن، هر كدام از شاخص­های حياتي ریوی را براي وي محاسبه و پيش­بيني نمود. به­عبارت ديگر ميزان متوسط هر شاخص استانداردي را كه براي فرد قابل تصور است، به­دست مي­دهد و لذا اين مقادير مبناي مقايسه­اي براي طبيعي يا غيرطبيعي بودن شاخص­های اندازه­گیری شده در فرد است.

Title: تزريق داخل پروستات توکسين بوتولينيوم-A (BTX-A): روش درمانی جديد برای هايپرپلازی خوش¬خيم پروستات

http://knh.shmu.ac.ir/index.php/site/article/view/179

مقدمه: در اين مطالعه، تأثير بالينی تزريق توکسين بوتولينيم-A در درمان هايپرپلازی خوش­خيم پروستات (BPH) مورد ارزيابی قرار گرفته است. مواد و روش­ها: در این مطالعه تعداد 55 بيمار مبتلا به BPH با سابقه درمان طبی ناموفق به­دلیل اینکه در معرض خطر بالایی برای جراحی قرار داشتند، تحت درمان با توکسين بوتولينيم-A در نقاط مختلف در زون ترانزيشنال قرار گرفتند. میزان آنتی­ژن مخصوص پروستات (PSA)، معیار بررسی علایم پروستات (Prostate system score)، شاخص کیفیت زندگی بیماران دارای مشکل ادراری، حجم پروستات، میزان باقی­مانده ادرای، میزان حداکثر جریان ادراری در تمام بیماران قبل، 3 و 6 ماه بعد از تزریق ثبت و مقایسه شده است. نتايج: میانگین سنی بیماران برابر با 5/2±78 سال بود. میانگین نمره علایم ادراری و کیفیت زندگی پس از 3 ماه به­صورت معناداری بهبود يافت. میزان سرعت جریان ادرار افزايش مشخص و معناداری را در 3 و 6 ماه پس از درمان نشان داد. حجم ادرار باقيمانده 3 ماه و 6 ماه بعد پس از تزريق به­طور معناداری کاهش يافت. ميزان PSA سرم از ng/ml 5/1±1/5 به ng/ml 9/0±7/2 در 3 ماه پس از تزريق کاهش يافت (05/0>P). حجم پروستات از ml 8±65 به ml 6±59 در عرض يک ماه و به ml 5±42 در عرض 3 ماه کاهش يافت (05/0>P). حداکثر اثر درمانی پس از يک هفته ديده شد. در انتهای 6 ماه پيگيری، 48 بيمار بهبود علایمشان را گزارش کردند و در 32 بيمار، درمان طبی قطع شد و فقط در 7 بيمار، علایم باقی ماند. بحث: تحقيق ما نشان داد که استفاده از تزريق داخل پروستات توکسين بوتولينيم-A (BTX-A) باعث کاهش حجم سريع پروستات و کاهش در مقاومت مجرا در بيماران با انسداد خروجی مثانه به­دليل بزرگی پروستات مي­شود و مي­تواند به­عنوان درمان برای انسداد ادراری مقاوم در بيماران با هايپرپلازی خوش­خيم پروستات که دارای خطر بالا جهت انجام جراحی هستند، در نظر گرفته شود. اين روش دارای حداقل ميزان عوارض است و حتی مي­توان از اين درمان به­عنوان يک درمان همراه در کانسر پروستات استفاده کرد.

Title: ارزیابی تأثیر سابقه¬ی فامیلی سرطان بر ابتلا به سرطان کولورکتال: یک مطالعه¬ی مورد شاهدی

http://knh.shmu.ac.ir/index.php/site/article/view/180

مقدمه: اگرچه افزایش خطر سرطان کولورکتال در افراد با سابقه­ی فامیلی مثبت در مطالعات گذشته مکرراً به اثبات رسیده است ولی اطلاعات بسیار محدودی در این زمینه در ایران گزارش شده است. هدف از این مطالعه تعیین ارتباط وجود سابقه­ی فامیلی سرطان و ابتلا به سرطان کولورکتال می­باشد. مواد و روش­ها: مطالعه حاضر به­صورت یک مطالعه مورد- شاهدی همسان نشده طراحی شده است. 393 مورد با تشخیص قطعی سرطان کولورکتال و 393 شاهد سالم که در یک طرح بررسی سلامتی شرکت کرده بودند به­طور تصادفی انتخاب شدند. اطلاعات مربوط به سابقه­ی فامیلی سرطان در موردها با استفاده از فرم­های ثبت سرطان و در شاهدها با مصاحبه با آنان توسط پرسنل بهداشتی آموزش دیده جمع­آوری شد. نسبت شانس مانتل- هانزل برای از بین بردن اثر مخدوش­کنندگی سن و جنس محاسبه گردید. نتایج: سابقه­ی فامیلی سرطان توسط 4/36% موردها و4/24% شاهدها گزارش گردید. خطر ابتلا به سرطان کولورکتال در افرادی که سابقه­ی ابتلا به سرطان در حداقل یکی از بستگان درجه­ی یک آن­ها گزارش گردیده بود در حدود 2 برابر افزایش می­یافت. نتایج مطالعه نشان داد که داشتن سابقه­ی فامیلی سرطان کولورکتال با احتمال 8/4 برابر (1/8-8/2:CI95/0) ابتلا به سرطان کولورکتال مصادف خواهد بود. نتیجه­گیری: بر اساس یافته­های این مطالعه می­توان چنین نتیجه­گیری کرد که داشتن سابقه­ی فامیلی سرطان با خطر ابتلا به سرطان کولورکتال همراه است. بر مبنای آن که، برنامه­ی معمول جهت غربالگری سرطان کولورکتال در ایران وجود ندارد، لذا توصیه می­شود بستگان درجه یک بیماران مبتلا به سرطان کولورکتال در اولویت برنامه­های غربالگری قرار بگیرند.

Title: مقايسه دو رويکرد يادگيري الکترونيکي سريع و يادگيري الکترونيکي مرسوم در آموزش ضمن خدمت دانشگاه علوم پزشکي تهران

http://knh.shmu.ac.ir/index.php/site/article/view/182

مقدمه: از آنجايي که تاکنون مطالعات معدودي به مقايسه يادگيري الکترونيکي سريع و مرسوم پرداخته­اند، مطالعه حاضر در سال 1387 با هدف مقايسه ميزان يادگيري، رضايت کارکنان تحت آموزش و زمان صرف­شده براي توليد دوره آموزشي با اين دو رويکرد در دانشگاه علوم پزشکي تهران انجام شد. مواد و روش­ها: در اين مطالعه مداخله­اي 100 نفر از کارکنان به­روش تصادفي بلوکي در دو گروه 50 نفري تحت آموزش با دوره آموزشي مبتني بر دو رويکرد، بررسی شدند. محتواي يادگيري الکترونيکي مرسوم توسط طراحي آموزشي آبشاري و نرم­افزار Macromedia flash و محتوای رويکرد يادگيري الکترونيکي سريع، با پردازش موازي سه مرحله تحليل، طراحي و تدوين و نرم­افزار Adobe Presenter تولید شد. داده­ها توسط پيش­آزمون، پس­آزمون، آزمون پايان دوره، پرسش­نامه­های خودارزيابيِ ميزان يادگيري و رضايت­سنجی کارکنان جمع­آوري شدند. نتايج: مقايسه نمره پيش­آزمون، پس­آزمون، آزمون پاياني و خودارزيابي کارکنان از ميزان يادگيريشان در دو گروه تفاوت معناداري نداشت. رضايت از محتواي آموزشي، شيوه آموزش، برنامه آموزش الکترونيکي و رضايت کلي در دو گروه تفاوت معناداري نداشت. زمان تدوين محتوا در رويکرد يادگيري الکترونيکي مرسوم، 141 روز کاري و در رويکرد سريع 24 روز کاري بود. نتيجه­گيري: نظر به عدم تفاوت دو رويکرد مذکور در ميزان يادگيري و رضايتمندي کارکنان و کاهش قابل توجه زمان تدوين دوره، در رويکرد يادگيري الکترونيکي سريع، استفاده از آن براي آموزش الکترونيکي کارکنان دانشگاه­ها، پيشنهاد مي­شود.

Title: بررسی آگاهی و نگرش ارایه¬دهندگان خدمات تنظیم خانواده در زمینه روش¬های پیش¬گیری از بارداری

http://knh.shmu.ac.ir/index.php/site/article/view/183

مقدمه: امروزه تنظيم خانواده به­عنوان يك اصل اساسي در زندگي افراد بشر در نظر گرفته مي‌شود. این مطالعه با هدف بررسی آگاهی و نگرش ارایه­دهندگان خدمات تنظیم خانواده در زمینه روش­های پیش­گیری از بارداری در شهرستان شاهرود در سال 1387 انجام شد. مواد و روش­ها: پژوهش از نوع مطالعات تحلیلی- مقطعي و كاربردي مي­باشد. در اين مطالعه پرسش­نامه­اي مشتمل بر 8 سؤال عمومي و 52 سؤال اختصاصي تهيه شده و جهت تكميل در اختيار ارایه­دهندگان قرار گرفت. جمعيت مورد مطالعه كليه ارایه­دهندگان خدمات تنظیم خانواده (162 نفر) بوده كه درخانه­های بهداشت، مراکز بهداشتی درمانی روستایی و شهری معاونت بهداشتي شاغل بودند. هم­بستگی بین متغیرها با استفاده از آزمون هم­بستگی پیرسون و مقایسه میانگین نمره بین گروه­ها با آزمون آنالیز واریانس انجام شده است. نتایج: در این مطالعه 152 نفر در مطالعه شرکت نموده که اکثریت آن­ها (7/73%) زن بودند. 3/74% ارایه­دهندگان بهورز، 9/7 درصد کاردان بهداشت خانواده، 6/2 درصد کارشناس بهداشت خانواده و 1/15 درصد ماما بودند. میانگین نمره آگاهی 7/2±9/17 از 25 بوده که حاکی از نمره آگاهی متوسط می­باشد. میانگین نمره نگرش 3/10±0/71 از 108 بوده که حاکی از نمره نگرش مثبت در افراد مورد مطالعه می­باشد. نتایج نشان داد رابطه معناداری بین آگاهی با جنس، شغل، مدرک تحصیلی، واحد خدمتی، سابقه کار و نوع روش پیش­گیری مورد استفاده توسط ارایه­کننده خدمت وجود دارد (001/0 P=). هم­چنین بین نگرش با جنس، واحد خدمتی، سابقه کار و آگاهی رابطه معناداری مشاهده نشد. نتیجه­گیری: با عنایت به آگاهی متوسط ارایه­دهندگان خدمات تنظیم خانواده و نگرش نسبتاً مثبت و عدم استفاده از روش­های پیش­گیری مطمئن در بین حدود یک سوم از آنان به نظر می­رسد برگزاری کلاس­های آموزشی در زمینه تنظیم خانواده برای کلیه رده­های ارایه­کننده خدمات می­تواند کمک شایانی در ارتقاء برنامه­های بهداشت مادران داشته باشد.

Title: بررسی تأثیر وضعیت انتخابی مادر در فاز فعال زایمان بر پیامد بارداری در زنان نخست¬زا

http://knh.shmu.ac.ir/index.php/site/article/view/184

مقدمه: ساعات پایانی بارداری انسان با انقباضات پرقدرت رحمی که موجب باز شدن دهانه رحم و بیرون راندن جنین از مجرای زایمان می­شود، مشخص می­گردد. وضعیت مادر در طول زایمان می­تواند بر روی انقباضات رحمی و هم­چنین عوامل روحی وی تأثیر بگذارد. در این مطالعه تأثیر وضعیت انتخابی مادر در مرحله زایمان بر پیامد بارداری مورد بررسی قرار گرفته است. مواد و روش­ها: پژوهش حاضر یک مطالعه تجربی است. در این مطالعه از میان زنان نخست­زای مراجعه­کننده به زایشگاه یا زهرا (س) تعداد 100 نفر که واجد شرایط مورد نظر در پژوهش بودند انتخاب و به­صورت تصادفی در دو گروه 50 نفری بررسی شدند. در گروه شاهد در بدو پذیرش (دیلاتاسیون 4 سانتیمتری) در اتاق درد به­صورت خوابیده در تخت مراقبت­های معمول را دریافت می­کردند ولی افراد گروه مداخله اجازه داشتند تا در هر وضعیتی که احساس راحتی بیش­تری می­کنند، قرار گیرند. اطلاعات از طریق فرم اطلاعاتی، ترازو، ساعت، معیار سنجش درد بورفورد، فرم ثبت مشاهدات و معاینات جمع­آوری با آزمون­های آماری t، کای- دو تجزیه و تحلیل شدند. نتایج: متوسط طول فاز فعال زایمان در گروه مداخله 2/142 دقیقه و در گروه شاهد 4/212 دقیقه، میانگین نمره درد در گروه مداخله 1/1±9/6 و در گروه دوم 2/1±1/8 گزارش شدند که اختلاف آن­ها معنادار می­باشد. درصد فراوانی مصرف اکسی توسین (8% در مقابل 64%) و هم­چنین میزان رضایت مادران از تجربه زایمان در گروه مداخله به­طور معناداری بیش­تر بود. میزان موارد غیرطبیعی صدای قلب جنین در گروه مداخله به­طور معناداری کم­تر از گروه شاهد بود. 76% واحدهای پژوهش در گروه مداخله در مقابل این سوال که آیا در حاملگی آینده وضعیت آزاد را انتخاب خواهند کرد، پاسخ مثبت دادند. نتیجه­گیری: وضعیت انتخابی مادر در مرحله فعال زایمان نه تنها هیچ­گونه اثر نامطلوبی بر پیامد بارداری ندارد بلکه بسیاری از نتایج بارداری را نیز بهبود می­دهد و می­توان آن را به­عنوان روشی مناسب در زایمان­های طبیعی و بدون عارضه به­کار برد.

Title: بررسي انتخاب نوع روش زايمان و عوامل مرتبط با آن در زنان باردار شهرستان مراغه

http://knh.shmu.ac.ir/index.php/site/article/view/185

مقدمه: در سال­هاي گذشته ميزان زايمان سزارين روند افزايشي داشته است. به­دليل عوارض جراحي، هزينه­هاي مالي، دوره اقامت و بستري شدن مادر در بيمارستان، زايمان طبيعي ارجحيت دارد. با اين حال در خواست و گرايش زنان به زايمان سزارين يکي از عوامل اساسي افزايش سزارين مي­باشد. هدف مطالعه حاضر، بررسي انتخاب نوع روش زايمان و دليل آن و تعيين برخي عوامل مرتبط با آن در زنان باردار شهرستان مراغه مي­باشد. مواد و روش­ها: در اين مطالعه توصیفی مقطعي 250 زن در سه مرکز بهداشتي- درماني شهرستان مراغه در شهريور ماه 1385 مورد مصاحبه قرار گرفتند. پرسش­نامه اين پژوهش شامل سؤالاتي براي كسب اطلاعات دموگرافيك، سابقه باروري، انتخاب نوع زايمان و دليل انتخاب بود. داده‌ها با استفاده از آزمون مجذور كاي و آزمون t مستقل در نرم­افزار SPSS تجزيه و تحليل گرديد. نتایج: از ميان 250 زن انتخاب شده 179 مورد (6/71%) زايمان طبيعي و 71 مورد (4/28%) زايمان سزارين را انتخاب نمودند. داشتن سابقه سزارين و محل مراجعه براي كنترل بارداري با انتخاب نوع زايمان ارتباط معناداري داشت (001/0P<). علت انتخاب زايمان سزارين به­ترتيب ترس از دردهاي زايماني، توصيه پزشك و تجربه سزارين قبلي بود. نتيجه­گيري: در صد انتخاب زايمان سزارين بالاست و برنامه­هاي مداخله­اي براي تغيير نگرش زنان نسبت به زايمان سزارين و افزايش آگاهي زنان در مورد زايمان بي­درد براي كاهش شيوع زايمان سزارين لازم است.

Title: بررسي کمي و کيفي پتانسيل بازيافت پسماندهای جامد شهر تهران

http://knh.shmu.ac.ir/index.php/site/article/view/186

مقدمه: بررسي ميزان و ترکيب اجزاء پسماندهاي شهری به­عنوان اطلاعات اساسي براي طراحي، بهره­برداري و بهينه­سازي سيستم­هاي مديريت پسماند مورد نياز است. هدف نهايي اين تحقيق بررسي کمي، کيفي و پتانسيل بازيافت پسماندهاي جامد شهر تهران به تفکيک مناطق 22 گانه آن مي­باشد. مواد و روش­ها: اين مطالعه در بازه زمانی 9 ماه ابتداي سال 1386 و در مناطق 22 گانه شهرداري تهران انجام شد. روش نمونه­برداري پسماندها به­صورت مرکب سيستماتيك بود. در اين مطالعه اجزاء خشک موجود در پسماندها، جداسازي و ابتدا با آزمون کلموگروف-اسمیرنوف طبیعی بودن توزيع ميانگين پسماندها و سپس براي بررسي وجود اختلاف معنادار بين پارامترهاي با توزیع نرمال، از آزمون آنالیز واریانس یک­طرفه و آزمون t و برای پارامترهاي با توزیع غيرنرمال از آزمون من­ویتنی و کروسکال والیس استفاده شد. نتايج: اجزاء خشك موجود در پسماندهاي مطالعه شده شامل کاغذ و مقوا 37%، پلاستيك 25%، فلزات 13%، منسوجات 8% و شیشه 4% بودند. بيش­ترين مقادير کاغذ و مقوا در آذر ماه، پلاستيک و فلزات در ارديبهشت ماه، منسوجات و شيشه در آبان­ماه و زایدات قابل دفن در خرداد ماه بودند. هم­چنين میانگین اجزاء خشک پسماندهای مورد مطالعه در ماه­های فصل پاییز نسبت به فصل تابستان دارای اختلاف معنادار می­باشند (05/0P<). نتيجه­گيري: پيشنهاد مي­شود كه برنامه تفكيك از مبدأ و راه­اندازي و ساماندهي صنايع بازيافت موجود در شهر تهران انجام شود كه علاوه بر كاهش حجم پسماند و كاهش هزينه­هاي مديريت پسماندهاي شهر تهران، موجب درآمدزايي و ايجاد اشتغال در اين بخش خواهد شد.

Title: بررسي شيوع و نحوه¬ی مصرف كندر در زنان باردار مراجعه¬كننده به مراكز بهداشتي درمانی مشهد

http://knh.shmu.ac.ir/index.php/site/article/view/187

مقدمه: به زنان باردار کندر بخورانيد، کودک هرگاه در شکم مادر با کندر تغذيه شود دلش قوی و عقلش زياد گردد (پيامبر گرامی اسلام (ص)). كندر (Frankincense) صمغ گياهي است كه از گونه­هاي مختلف گياه Boswellia به­دست مي­آيد. در طب سنتي و پزشكي اسلامي از كندر به­عنوان ماده­ای كه اثر تقويت هوش و حافظه دارد نام برده شده است. مهم­ترين جايگاه يادگيري و حافظه در مغز، هيپوكامپ است، احتمالاً عصاره كندر با تأثير بر اين ناحيه موجب افزايش انتقال پيام­ها و تقويت حافظه مي­شود. با توجه به استفاده گسترده از توصيه­های طب سنتی و اسلامی در کشور، در مطالعه­ی حاضر، وضعيت استفاده از کندر در دوران بارداری در زنان تحت پوشش مراکز بهداشتی در مانی شهرستان مشهد در سال 1386-1385 بررسی شده است. مواد و روش­ها: در اين پژوهش مقطعی تعداد 900 زن باردار مراجعه­کننده به مراکز بهداشتی درمانی مورد مصاحبه قرار گرفتند. افراد واجد شرایط طی نمونه­گیری چند مرحله­ای انتخاب شدند. سن بارداري در زنان باردار مورد پژوهش بين 4 تا 41 هفته و ميانگين آن 2/26 هفته بود. جهت پردازش داده­ها از نرم­افزارSPSS  استفاده شد. نتایج: 263 نفر (2/29 درصد) از مادران به­منظور با هوش شدن كودك از كندر استفاده كرده بودند. ميزان مصرف کندر از يك نوبت در طي بارداري تا مصرف روزانه در کل دوران بارداري متفاوت و به­طور متوسط 2/74 روز از دوران بارداری بود. 8/39 درصد از مادران استفاده­كننده از کندر داراي تحصيلات زير ديپلم و 2/60 درصد داراي تحصيلات ديپلم و بالاتر بودند. در 6/94 درصد از مادران مصرف­كننده هيچ عارضه­اي در بارداري مشاهده نشد و تنها در 4/5 درصد از مادران تهوع يا ساير عوارض جزيي همراه با مصرف کندر گزارش گرديد. نتيجه­گيری: با توجه به اهميت يادگيری و نقش تعيين­كننده قواى ذهنى و حافظه در زندگى و موفقيت انسان، تحقيقات تكميلي براي تعيين ميزان تأثير آن بر بهره هوشي كودكان متولد شده از مادران مصرف­كننده كندر و مقايسه آن با ساير كودكان توصيه مي­شود.
